# Insertion Pool Sequencing for Insertional Mutant Analysis in Complex Host‐Microbe Interactions

**DOI:** 10.1002/cppb.20097

**Published:** 2019-07-03

**Authors:** Simon Uhse, Florian G. Pflug, Arndt von Haeseler, Armin Djamei

**Affiliations:** ^1^ Gregor Mendel Institute (GMI), Austrian Academy of Sciences Vienna BioCenter (VBC) Vienna Austria; ^2^ Center for Integrative Bioinformatics Vienna (CIBIV), Joint Institute of the University of Vienna and Medical University of Vienna Max F. Perutz Laboratories (MFPL) Vienna Austria; ^3^ Bioinformatics and Computational Biology, Faculty of Computer Science University of Vienna Vienna Austria; ^4^ Leibniz Institute of Plant Genetics and Crop Plant Research (IPK) Gatersleben Germany

**Keywords:** fungal genomics, maize, plant genomics, plant‐microbe interactions, unique molecular identifiers, *Ustilago maydis*, virulence factors

## Abstract

Insertional mutant libraries of microorganisms can be applied in negative depletion screens to decipher gene functions. Because of underrepresentation in colonized tissue, one major bottleneck is analysis of species that colonize hosts. To overcome this, we developed insertion pool sequencing (iPool‐Seq). iPool‐Seq allows direct analysis of colonized tissue due to high specificity for insertional mutant cassettes. Here, we describe detailed protocols for infection as well as genomic DNA extraction to study the interaction between the corn smut fungus *Ustilago maydis* and its host maize. In addition, we provide protocols for library preparation and bioinformatic data analysis that are applicable to any host‐microbe interaction system. © 2019 The Authors. This is an open access article under the terms of the Creative Commons Attribution License, which permits use, distribution and reproduction in any medium, provided the original work is properly cited.

## INTRODUCTION

Insertional mutagenesis has been used in fungi, including baker's yeast and *Cryptococcus neoformans*, to decipher gene functions (Giaever et al., 2002; Idnurm, Reedy, Nussbaum, & Heitman, 2004; Winzeler et al., 1999). Negative depletion screening in combination with insertional mutagenesis libraries is a powerful approach to decipher gene functions under a certain condition, e.g., host infection (Jeon et al., 2007). Recent advances in massive parallel sequencing allow for large‐scale approaches in bacteria that permit analysis of larger pools of insertional mutants (Gawronski, Wong, Giannoukos, Ward, & Akerley, 2009; van Opijnen, Bodi, & Camilli, 2009). Here, we describe the insertion pool sequencing (iPool‐Seq) pipeline that we recently established with the *Ustilago maydis*–*Zea mays* interaction (Uhse et al., 2018).

*U. maydis* is a smut fungus that colonizes and overcomes the immunity of the crop plant maize (Kamper et al., 2006). Many molecular and genetic tools are available for *U. maydis*, and therefore, the fungus is an important model organism in the field of plant‐microbe interactions (Lanver et al., 2017). Especially useful for generation of an insertional mutagenesis library is the availability of a solopathogenic *U. maydis* strain that is haploid and capable of infecting (Kamper et al., 2006). Many plant pathogens, including *U. maydis*, rely on the versatile repertoire of effector genes that mediate and shape the interaction with the host plant. The majority of predicted *U. maydis* effector genes are unstudied, and it is unclear if and to what extent they contribute to virulence (Kamper et al., 2006). To gain insights about the effector repertoire of *U. maydis*, we generated an insertional mutagenesis library and employed it in a negative depletion screen during infection of maize to elucidate novel virulence factors in the fungus.

Transformation of insertional cassettes via homologous recombination is well established in *U. maydis* and has been used successfully to delete clusters of predicted effector genes (Kamper et al., 2006). We created an insertional mutant library for *U. maydis* via homologous recombination of a selectable marker conferring resistance to hygromycin. Next, we established the iPool‐Seq workflow based on this library, allowing for controlled insertional mutagenesis at loci of predicted effectors that are likely to contribute to the virulence of *U. maydis* (Uhse et al., 2018). All newly generated *U. maydis* mutants were verified for deletion of the targeted effector genes via PCR on cultures and on extracted genomic DNA (gDNA). Eventually, the library comprised three independent replicates for each insertional mutant, with 195 putative virulence factor mutants for *U. maydis* in total. We used this library to conduct a negative depletion screen by infection of the host plant maize and subsequent analysis of the input and output compositions. The input, i.e., the gDNA of the mutant pool before the infection, and the output, i.e., the gDNA of the infected host material, were prepared, deep‐sequenced, and bioinformatically analyzed.

iPool‐Seq was performed on the entire library of 195 insertional mutants. The method was highly selective for reads from *U. maydis* insertional mutant loci, yielding >75% informative reads for input and output samples. Moreover, we identified 28 reproducibly and significantly depleted mutants from next‐generation sequencing (NGS) reads after infection in the maize Early Golden Bantam. Several of the mutants that we found with iPool‐Seq were previously shown to be virulence factors. For instance, mutants of the *U. maydis* effectors Pep1, ApB73, and, more recently, Cce1 displayed severe virulence defects based on classical disease ratings (Doehlemann et al., 2009; Seitner, Uhse, Gallei, & Djamei, 2018; Stirnberg & Djamei, 2016). The confirmation of these known virulence factors by iPool‐Seq indicates that iPool‐Seq yields reliable results for depleted mutants. To further strengthen this finding, we tested three potential virulence factors identified by iPool‐Seq and were able to show a virulence phenotype for these mutants based on classical disease ratings in comparison to the wildtype solopathogenic strain SG200 (Uhse et al., 2018).

**Figure 1:**
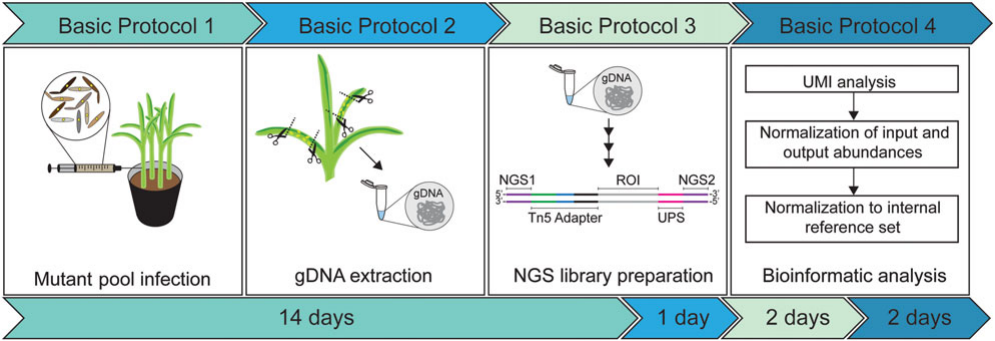
Overview of the iPool‐Seq pipeline. The pipeline contains four parts, which can be finished sequentially in ∼20 days. gDNA, genomic DNA; NGS, next‐generation sequencing; UMI, unique molecular identifier.

The protocols described here aim to make the full potential of iPool‐Seq accessible to the larger scientific community. The iPool‐Seq workflow, from infection to sequence analysis, is divided into four parts (Fig. 1): Basic Protocol 1 describes the process of infection of the host plant maize with insertional mutant pools. Basic Protocol 2 describes the extraction of gDNA from input samples before infection and from output samples after infection. Both Basic Protocol 1 and Basic Protocol 2 were established for *the U. maydis*–*Z. mays* pathosystem and might require adaptation when applied to other host‐microbial interaction systems. In contrast, Basic Protocols 3 and 4 are applicable to any host‐microbial interaction system: Basic Protocol 3 describes the NGS library preparation in detail, and Basic Protocol 4 details bioinformatic analysis of the sequencing results for the input and output libraries, with the goal of detecting changes in the virulence of particular insertional mutants compared to a reference set of neutral controls.

## Basic Protocol 1 *U. MAYDIS* INSERTIONAL MUTANT POOL INFECTION IN MAIZE

For generation of a negatively depleted output, the insertional mutant library of *U. maydis* must be raised, pooled, and infected into its host maize. The following protocol describes the processes of infection and of harvest of the infected maize tissue.

*NOTE*: Repeat the procedure for a total of three biological replicates.

### Materials

Soil [4:1 mixture of standard potting soil (Einheitserde Werkverband e.V.) with perlite (Granuflor)]
Early Golden Bantam (EGB) maize seeds
Nematode (Biohelp) solution (1 g in 3 L water)
Cryopreserved *U. maydis* insertional mutant library (strain SG200 background genotype; transformed via homologous recombination; Kamper et al., 2006; Uhse et al., 2018)
Potato dextrose–agar plates containing 200 µg/ml hygromycin (see recipe)
YepsLight liquid medium (see recipe)
Double‐distilled water
0.05% (v/v) Tween‐20 (Sigma‐Aldrich) in double‐distilled water
Liquid nitrogen

12‐cm‐diameter round pots
Phytochamber
5‐ml glass pipets
48‐deep‐well liquid culture plates (5‐ml well volume, UCT)
28°C incubator‐shaker
15‐ and 50‐ml Falcon tubes
Rotator
Photometer/plate reader
500‐ml centrifuge tube
Standard tabletop centrifuge
1‐ml syringe (B. Braun)
0.45‐mm‐diameter needle (25‐mm long, B. Braun)
Scissors
1‐L beaker (Duran)
Magnetic stirrer (IKA) and stir bar
Mortar and pestle
Metal spatula
Laboratory Mixer Mill MM 200 (Retsch) and compatible container
8‐mm‐diameter metal balls (Kugel‐Pompel)

*NOTE*: All reagents, consumables, and equipment coming into contact with living *U. maydis* axenic culture cells must be sterile. Working in a laminar flow hood is recommended, if possible.

### Potting of maize

1 Distribute soil in 12‐cm‐diameter round pots and water pots sufficiently. Seed five EGB maize seeds per pot for a total of >100 seeds per insertional mutant pool replicate. Treat each pot with 100 ml nematode solution for pest control.
2 Grow maize in a phytochamber under the following conditions: 14‐hr/10‐hr light/dark cycle at 28°C/20°C with a total light intensity of 183.21 µmol m^−^ s^−1^.
  After 7 days, maize seedlings are ready for infection with the insertional mutant pool.

### Growth of U. maydis insertional mutant library

3 Distribute cryopreserved *U. maydis* insertional mutant library strains on potato dextrose–agar plates containing 200 µg/ml hygromycin using 5‐ml glass pipets. Keep individual mutant strains separate by streaking ≤8 strains on designated sectors per plate. Grow at 28°C for 2 to 3 days in the dark.
4 Inoculate each strain in 2 ml YepsLight liquid medium per well in 48‐deep‐well liquid culture plates. Grow infection pre‐culture overnight in a 28°C incubator‐shaker with agitation at 180 rpm.
5 Inoculate 5 ml YepsLight per 15‐ml Falcon tube with pre‐culture at a 1:3000 ratio to form infection main cultures. Grow overnight (for 15 hr) at 28°C in a rotator at 20 rpm.
6 Measure optical density at 600 nm in a photometer/plate reader for each individual mutant strain infection main culture. Adjust the amount of culture to achieve an optical density between 0.6 and 1 for each strain and pool equal volumes of cultures of all mutant strains in a 500‐ml centrifuge tube.
7 Centrifuge 10 min at 2000 × *g* and discard supernatant by decanting, ensuring removal of all supernatant. Resuspend pellet in double‐distilled water to an optical density at 600 nm of 1 by pipetting up and down.

### Infection of U. maydis insertional mutant library in maize seedlings

8 Using a 1‐ml syringe and a 0.45‐mm‐diameter needle, inject ∼250 µl pooled infection culture into 7‐day‐old EGB maize seedlings (see step 2) that display three juvenile leaves. Make sure to infect maize seedlings in the center of the leaf whirl by piercing the stem halfway. Infect a total of >100 maize plants with the pool.
9 For the input control, centrifuge 10 ml pooled infection culture in a 15‐ml Falcon tube for 1 min at 10,000 × *g*. Discard supernatant and store pellet at −70°C until isolation of gDNA from the input sample (see Basic Protocol 2).

### Harvest of infected maize tissue

10 Grow infected maize seedlings from step 8 for another 7 days in a phytochamber with a 14‐hr/10‐hr light/dark cycle at 28°C/20°C with a total light intensity of 183.21 µmol m^−2^ s^−1^.
11 Harvest infected second and third maize leaves at a 1‐cm distance from the infection site with scissors, making sure to restrict the harvested material to infected tissue. Wash harvested tissue in 0.05% Tween‐20 in double‐distilled water in a 1‐L beaker by stirring on a magnetic stirrer with a magnetic stir bar at 200 rpm for 5 min. Subsequently, wash leaves twice in double‐distilled water. Air‐dry wet leaves at room temperature before cryopreservation (see steps 12 to 14).
  The wash steps facilitate removal of any remaining dead insertional mutants located on the leaf epidermis.
12 Crush dry infected maize tissue in liquid nitrogen with a mortar and pestle.
  From now on, cool down all consumables and equipment with liquid nitrogen and avoid thawing of the maize tissue to ensure gDNA integrity.
13 Using a metal spatula, transfer crushed material into a container compatible with the Laboratory Mixer Mill MM 200 and add three 8‐mm‐diameter metal balls. Mill samples at 25 Hz for 90 sec. Cool mill container in liquid nitrogen for 60 sec and repeat milling step.
14 Using a metal spatula, transfer milled maize powder into a 50‐ml Falcon tube and store at −70°C until gDNA extraction from the output sample (see Basic Protocol 2).

## Basic Protocol 2 gDNA EXTRACTION FROM MUTANT POOL BEFORE AND AFTER INFECTION OF MAIZE

The starting material for the iPool‐Seq Illumina library preparation is gDNA (Basic Protocol 1). Firstly, gDNA from the input library is required to analyze the composition of the initial insertional mutants. Secondly, the gDNA of the infected material is required to obtain insights about the insertional mutant pool composition after infection. *U. maydis* gDNA extraction is based on a protocol established in yeast that was adapted for iPool‐Seq (Hoffman & Winston, 1987).

### Materials

Glass beads (450 to 600 µM, B. Braun)
Input pellet (10‐ml pellet of pooled mutants before infection, stored at −70°C; see Basic Protocol 1, step 9)
TE‐equilibrated phenol/chloroform/isoamyl alcohol [25:24:1 (v/v/v), pH 7.5 to 8, Carl Roth; store at 4°C in the dark]
Ustilago lysis buffer (see recipe)
80% (v/v) and 100% ethanol (p.a.)
1× TE containing 20 μg/ml RNase A (Thermo Fisher Scientific, EN0531; store at −20°C)
Homogenized infected maize tissue (output, stored at −70°C; see Basic Protocol 1, step 14)
Isopropanol (p.a.)

VXR basic Vibrax (IKA) or equivalent
Refrigerated tabletop centrifuge, 4°C
ThermoMixer C (Eppendorf)
Scale
7‐ml Precellys tube (Bertin)
Precellys Evolution bead mill (Bertin)
5‐ml Eppendorf tubes

*NOTE*: Conduct all steps on ice and pre‐cool consumables and equipment to 4°C.

*CAUTION*: Phenol/chloroform is highly toxic and volatile. Take protective measures when working with phenol/chloroform, including working in a laminar flow hood. Handle phenol‐contaminated waste appropriately.

*CAUTION*: Store the ethanol and isopropanol at 5°C to 30°C in a safety cabinet for flammable liquids.

### gDNA extraction from input

1 Add ∼200 µl glass beads to the input pellet (see Basic Protocol 1, step 9). Add 500 µl TE‐equilibrated phenol/chloroform/isoamyl alcohol and 400 µl Ustilago lysis buffer.
2 Vortex mixture at 1500 rpm for 15 min at room temperature in a VXR basic Vibrax or equivalent device.
3 Centrifuge 30 min at 13,000 × *g*, 4°C.
4 In the meantime, prepare a 1.5‐ml Eppendorf tube containing 1 ml of 100% ethanol.
5 Add 400 µl of upper, aqueous layer from step 3 to the tube from step 4 and mix vigorously by vortexing for 30 sec.
6 Incubate mixture for >1 hr at −20°C to improve gDNA precipitation.
  Stopping point: Store the mixture overnight at −20°C.
7 Centrifuge 5 min at 13,000 × *g*, 4°C.
8 Wash gDNA pellet with 1 ml of 80% ethanol. Invert tube several times and centrifuge 5 min at 13,000 × *g*, 4°C.
9 Remove supernatant carefully by pipetting, briefly spin down tube, and remove residual supernatant.
10 Add 30 µl of 1× TE containing 20 μg/ml RNase A.
11 Incubate for 15 min at 55°C on a ThermoMixer C with agitation at 800 rpm and with an open lid.
  This step allows for evaporation of residual ethanol.
12 Store extracted input gDNA at −20°C until library preparation (see Basic Protocol 3).

### gDNA extraction from output

13 Weigh 1 g homogenized infected maize tissue (output; see Basic Protocol 1, step 14) and transfer into a 7‐ml Precellys tube.
14 Add ∼1000 µl glass beads to output. Add 2.5 ml TE‐equilibrated phenol/chloroform/isoamyl alcohol and 2 ml Ustilago lysis buffer.
15 Vortex mixture at 5000 rpm for 30 sec at room temperature in a Precellys Evolution bead mill. Transfer mixture into 5‐ml Eppendorf tubes.
16 Centrifuge 30 min at 13,000 × *g*, 4°C.
17 In the meantime, prepare a 5‐ml Eppendorf tube containing 2.2 ml isopropanol.
18 Add 1.5 ml of upper, aqueous layer from step 16 to the tube from step 17 and mix vigorously by vortexing for 30 sec.
19 Incubate mixture for >1 hr at −20°C to improve gDNA precipitation.
  Stopping point: Store the mixture overnight at −20°C.
20 Centrifuge 5 min at 13,000 × *g*, 4°C.
21 Wash gDNA pellet with 1 ml of 80% ethanol. Invert tube several times and centrifuge 5 min at 13,000 × *g*, 4°C.
22 Remove supernatant carefully, briefly spin down tube, and remove residual supernatant.
23 Add 150 µl of 1× TE containing 20 μg/ml RNase A.
24 Incubate for 15 min at 55°C on a ThermoMixer C with agitation at 800 rpm and with an open lid.
  As in step 11, this step allows for evaporation of residual ethanol.
25 Store extracted output gDNA at −20°C until library preparation (see Basic Protocol 3).

## Basic Protocol 3 ILLUMINA SEQUENCING LIBRARY PREPARATION USING gDNA FROM INSERTIONAL MUTANT POOLS

The purified gDNA from the insertional mutant library input and output (Basic Protocol 2) is further processed via an iPool‐Seq library preparation protocol to obtain Illumina sequencing–compatible libraries. The protocol is optimized for specific enrichment of insertion mutant flanks and high double‐stranded DNA (dsDNA) yields, enabling library preparation directly from infected host tissue.

*NOTE*: Check the dsDNA concentration after each step to ensure successful preparation. We recommend quantification via a fluorescence assay, e.g., PicoGreen.

### Materials

Oligonucleotides: 100 µM Adapters P1 and P2 and 5 µM PCR1‐F, PCR1‐R, PCR2‐F, and PCR2‐R (see Table 1)
Reassociation buffer (see recipe)
Purified Tn5 transposase [1 µg/µl, prepared according to prior protocol (Picelli et al., 2014); store at −70°C in aliquots for ≤12 months]
100% glycerol
Input and output gDNA (see Basic Protocol 2, steps 12 and 25, respectively)
5× TAPS buffer (see recipe)
Nuclease‐free water
SPRI magnetic beads (e.g., Agencourt AMPure XP beads, Beckman Coulter; store at 4°C)
Phusion High‐Fidelity DNA Polymerase, with 5× Phusion HF Buffer (New England Biolabs, M0530; store at −20°C)
10 mM dNTPs (New England Biolabs, N04472; store at −20°C)
Dynabeads M‐270 Streptavidin
1× and 2× B&W buffers (see recipes)

**Table 1:** Oligonucleotides Used for Adapters, Specific PCR1 and PCR2, and Illumina Sequencing in Basic Protocol 3

Oligonucleotide	Sequence^a^
Adapter P1^b^	5′‐CACGACGCTCTTCCGATCTNNNNNNNNNNNNAGATGTGTATAAGAGACAG‐3′
Adapter P2	5′‐[phos]CTGTCTCTTATACACATC[3InvdT]‐3′
PCR1‐R^c^^,^ d	5′‐[BioTEG]CCAGATGTCCTGTGGTATCCTGTG‐3′
PCR1‐F	5′‐GAGATCTACACTCTTTCCCTACACGACGCTCTTCCGATC‐3′
PCR2‐F	5′‐AATGATACGGCGACCACCGAGATCTACACTCTTTCCCTACAC‐3′
PCR2‐R^d^	5′‐CAAGCAGAAGACGGCATACGAGATNNNNNNGTGACTGGAGTTCAGACGTGTG CTCTTCCGATCTCCTGTGGTATCCTGTGGCG‐3′
Illumina Rd1^e^	5′‐ACACTCTTTCCCTACACGACGCTCTTCCGATCT‐3′
Illumina Rd2^e^	5′‐GTGACTGGAGTTCAGACGTGTGCTCTTCCGATCT‐3′

a Depending on the library of insertional mutants that is screened, these sequences may potentially need to be adjusted.

b The 12 Ns in Adapter P1 constitute the UMI and should be random.

c The 6 Ns in PCR2‐R constitute the (single‐index) library multiplexing barcode.

d With PCR1‐R and PCR2‐R as listed, enrichment of insertion mutant flanks is specific for the sequence 5′‐CCAGATGTCCTGTGGTATCCTGTGGCG‐3′, and read2 will start with the underlined part of PCR2‐R.

e Illumina Rd1 and Rd2 are the standard Illumina TruSeq sequencing primers.

PCR tubes
Thermocycler
Gel electrophoresis chamber or fragment analyzer
1.5‐ml DNA LoBind tubes (Eppendorf)
Magnetic stand
Rotator

Additional reagents and equipment for gel electrophoresis (see Current Protocols article; Gallagher, 2012)

### Tn5 fragmentation of input and output gDNA

1 Combine the following in a PCR tube to 100 µl total volume and mix well by pipetting up and down:
  - 25 µl of 100 µM Adapter P1
  - 25 µl of 100 µM Adapter P2
  - 50 µl reassociation buffer.
  Perform primer annealing in a thermocycler starting from 90°C, with a 1°C decrement per minute.
2 Combine the following in a PCR tube to 100 µl total volume and mix well by pipetting up and down:
  - 25 µl purified Tn5 transposase
  - 25 µl annealed adapters (see step 1)
  - 50 µl of 100% glycerol.
3 Incubate for 30 min at 37°C in a thermocycler.
4 Combine the following in separate PCR tubes and mix well by pipetting up and down:
  - 250 ng input or output gDNA
  - Tn5 transposase loaded with adapters (see step 3) to a final concentration of 150 ng/μl
  - 4 µl of 5× TAPS buffer and
  - Nuclease‐free water to 20 µl.
    CAUTION: TAPS buffer contains dimethylformamide (DMF), which is toxic and volatile. Take appropriate safety measures and work in a laminar flow hood.
    Prepare separate reactions for the input and output and use 250 ng per PCR reaction. For the Tn5 fragmentation, we recommend total amounts of 500 ng for the input gDNA and 10 × 1 µg for the output gDNA for a first trial. Upscaling is recommended if final read coverage of insertional mutants and unique molecular identifier (UMI) diversity is low.
5 Incubate reaction for 10 min at 55°C in a thermocycler.
6 Clean fragmentations with SPRI magnetic beads, e.g., Agencourt AMPure XP beads, according to the manufacturer's protocol.
  We recommend gDNA purification with SPRI beads to minimize DNA loss. A ratio of 1.5× SPRI beads to DNA yields good results. Size selection is possible but not recommended. Column‐based purification systems can be used instead of SPRI magnetic beads but will most likely provide lower DNA recovery yields and thus less diverse sequencing libraries.
7 Ensure fragmentation success via gel electrophoresis or using a fragment analyzer.
8 Determine dsDNA concentration for subsequent PCR.
  We recommend quantification via a fluorescence assay, e.g., PicoGreen, and a minimal final concentration of 20 ng/µl.
  Stopping point: Store the fragmented DNA at −20°C until specific PCR.

### Specific PCR of mutant cassette genome junctions

9 Combine the following in a PCR tube and mix well by pipetting up and down.
  - 5 µl of 5× Phusion HF Buffer
  - 1 µl of 10 mM dNTPs
  - 1 µl of 5 µM PCR1‐F
  - 1 µl of 5 µM PCR1‐R
  - 0.5 µl Phusion High‐Fidelity DNA Polymerase
  - 250 ng fragmented and cleaned gDNA (see step 6)
  - Nuclease‐free water to 25 µl.
10 Run the following PCR program in a thermocycler:

**Table:** 

Initial step:	1 min	95°C	(initial denaturation)
15 cycles:	15 sec	95°C	(denaturation)
	15 sec	65°C	(annealing)
	30 sec	72°C	(elongation)
Final step:	1 min	72°C	(final elongation).
11 Clean PCR reactions with SPRI magnetic beads according to the manufacturer's protocol.
12 Pool clean eluates. Optional: Check dsDNA concentration prior to streptavidin enrichment (see steps 13 to 19).
  Stopping point: Store the cleaned specific PCR fragments at −20°C until streptavidin enrichment.

### Streptavidin enrichment

13 For each input and output sample, wash 30 µl Dynabeads M‐270 Streptavidin in 200 µl of 1× B&W buffer in a 1.5‐ml DNA LoBind tube on a magnetic stand.
14 Repeat washing step three additional times.
15 Resuspend beads in 2× B&W buffer, matching the volume of the clean input and output PCR1 eluates.
16 Pool eluates and beads and allow for enrichment of biotinylated PCR amplicons by rotation at room temperature for 15 min.
17 Place tubes in a magnetic stand for 1 min and discard supernatant.
18 Wash beads with 200 µl of 1× B&W buffer while the tubes remain in the magnetic stand.
19 Repeat washing step three additional times. Resuspend beads in 34 µl nuclease‐free water and proceed with nested PCR (see steps 20 and 21).
  Stopping point: Store the enriched PCR fragments at −20°C until nested PCR.

### Nested PCR of enriched fragments

20 Combine the following in PCR tubes to 50 µl total volume and mix well by pipetting up and down:
  - 34 µl nuclease‐free water containing beads (see step 19)
  - 10 µl of 5× Phusion HF Buffer
  - 1 µl of 10 mM dNTPs
  - 2 µl of 5 µM PCR2‐F
  - 2 µl of 5 µM PCR2‐R
  - 1 µl Phusion High‐Fidelity DNA Polymerase.
21 Run the following PCR program in a thermocycler:

**Table:** 

Initial step:	1 min	95°C	(initial denaturation)
15 cycles:	15 sec	95°C	(denaturation)
	15 sec	65°C	(annealing)
	30 sec	72°C	(elongation)
Final step:	1 min	72°C	(final elongation).
22 Place tubes in a magnetic stand for 1 min and transfer supernatant containing PCR2 amplicons into fresh PCR tubes.
23 Clean PCR reactions with SPRI magnetic beads according to the manufacturer's protocol.
24 Check dsDNA concentration and proceed with Illumina sequencing (see Basic Protocol 4).
  We recommend a minimal final dsDNA concentration of 0.1 ng/µl to enable Illumina sequencing. Moreover, we suggest quality control of iPool‐Seq library preparation via qPCR and fragment length analysis prior to Illumina sequencing. We sequence the iPool‐Seq libraries on a MiSeq Illumina platform with 75PE conditions.
  IMPORTANT NOTE: The standard Illumina Nextera sequencing primers are not compatible with our Tn5 adapter and will interfere with sequencing if present. Instead, Illumina TruSeq sequencing primers (Table 1, Illumina Rd1 and Rd2) must be used.

## Basic Protocol 4 BIOINFORMATIC ANALYSIS

This protocol describes how insertion pool data are analyzed, using the pipeline that we developed (available at http://www.cibiv.at/software/ipoolseq‐pipeline), to find insertional mutants with significantly increased or decreased virulence. Virulence is measured as the abundance of a deletional mutant in the post‐infection output pool relative to the pre‐infection input and is compared to the virulence of a reference set of known neutral mutants to find significant deviations from neutral behavior.

*NOTE*: In the following, commands intended to be entered on a Unix‐style terminal, either directly on a Linux machine or via SSH, are printed in a monospaced font. Outside of such commands, filenames are printed monospaced and underlined. Placeholders for names of experiments or libraries that have to be supplied by the user are printed in *italic*.

*NOTE*: iPool‐Seq pipeline is based on the workflow engine “snakemake” (Köster & Rahmann, 2012). It is thus not strictly necessary to proceed step by step; in particular, jumping to step 13 directly after adding all required data in step 6 will cause all intermediate steps to be executed automatically. However, given that it is good practice to check the results of key intermediate steps (like mapping and KO assignment) for validity before proceeding, we recommend following the steps outlined below and also performing the checks and validations suggested in the Troubleshooting section for each individual step.

### Materials

Workstation running Linux or Windows 10 with Windows Subsystem for Linux (WSL), with 64‐bit CPU and 8 GB or more of RAM and with free disk space ≥5 times size of raw sequencing data
iPool‐Seq analysis pipeline (http://www.cibiv.at/software/ipoolseq‐pipeline)
Reference genome of *U. maydis* in FASTA format
FASTA file containing sequences at 5′ end (named “5p”) and 3′ end (named “3p”) of knockout (KO) cassette
List of deletional mutants of *U. maydis* as GFF2 file listing KO cassette insertion positions
Single BAM file containing unmapped sequencing reads or two separate compressed FASTQ files (one for read1 and one for read2) for each sequenced library (prepared according to Basic Protocol 3)
Web browser
PDF viewer

*NOTE*: For the FASTA file describing the KO cassette, the sequences must reflect the part of read2 that overlaps with the cassette, i.e., start with the underlined part of PCR2‐R (Table 1) and extend up to the end of the cassette. See the (included) list of deletional mutants used by Uhse et al. (2018) cfg/Uhse_et_al.2018/cassette.fa for an example.

*NOTE*: For the list of deletional mutants, each entry must carry ≥2 two tags: a unique “Name” and a flag “Neutral” with value 0 or 1 that decides whether a particular deletion strain is included in the reference set of assumed neutral deletions. See the (included) list of deletional mutants used by Uhse et al. (2018) cfg/Uhse_et_al.2018/knockouts.gff for an example.

### Installing the iPool‐Seq pipeline

1 On a workstation running Linux or Windows 10 with WSL, download and install iPool‐Seq analysis pipeline by executing
VER=latest‐release
URL=http://github.com/Cibiv/ipoolseq‐pipeline
curl ‐L ‐O $URL/archive/$VER.tar.gz
tar xzf $VER.tar.gz
cd ipoolseq‐pipeline‐$VER
  You can also use a web browser to download the latest release from http://github.com/Cibiv/ipoolseq‐pipeline/releases. Then, change into the directory containing the downloaded file in a terminal window and continue with the tar command to unpack the archive.
2 Install and activate our Bioconda (http://bioconda.github.io; Grüning et al., 2018) environment, containing all software packages required by pipeline, with
./install‐environment.sh
source ./environment/bin/activate
  Should environment.tar.gz fail to download, download that file from http://github.com/Cibiv/ipoolseq‐pipeline manually and place it in the folder containing the pipeline.
  IMPORTANT NOTE: The environment must be re‐activated (but not re‐created) whenever you open a new terminal window.
3 Optional: Test for successful installation of library by re‐analyzing one of the replicates of Uhse et al. (2018). To download the raw sequencing data for replicate A1 (data/Uhse_et_al.2018/expA.r1‐in.bam and expA.r1‐out.bam) and to run the differential virulence analysis, do
snakemake data/Uhse_et_al.2018/expA.r1.dv.tab
  Afterward, data/Uhse_et_al.2018/expA.r1.dv.tab contains a table listing the differential virulence analysis results for all deletional mutants (see Table 3 for a list of columns), and an accompanying HTML report is written to data/Uhse_et_al.2018/expA.r1.dv.html. Note that although the pipeline discussed here uses the same approach as the pipeline used by Uhse et al., it differs in some details and does not cover combining data from multiple replicates. The re‐analysis thus cannot be expected to reproduce the published results exactly.

### Adding the reference genome, cassette file, KO list, and libraries

4 Pick a name (e.g., *your_design*) for the experimental design (including the reference genome and the list of KO cassette insertions) and create two folders with
mkdir ‐p data/*your_design*

mkdir ‐p cfg/*your_design*

5 Copy the reference genome of *U. maydis* in FASTA format to cfg/*your_design*/reference.fa, the FASTA file containing the sequences at 5′ end (named “5p”) and 3′ end (named “3p”) of the KO cassette to cfg/*your_design*/cassette.fa , and the list of deletional mutants of *U. maydis* as a GFF2 file listing KO cassette insertion positions to cfg/*your_design*/knockouts.gff.
6 For a single replicate (named *your_replicate* here, but it can have an arbitrary name), which always consists of two libraries, name pre‐infection input pool *your_replicate*‐in and post‐infection output pool *your_replicate*‐out. For each of those two libraries (in the following, referred to as *your_lib*, which thus stands for either *your_replicate*‐in or *your_replicate*‐out), place raw paired‐end sequencing data either into data/*your_design*/*your_lib*.bam as a single BAM file containing unmapped sequencing reads or into data/*your_design*/*your_lib*.1.fq.gz (read1) and data/*your_design*/*your_lib*.2.fq.gz (read2) as two separate compressed FASTQ files.
  This naming scheme ensures that the pipeline knows which reference genome and KO list belong to a particular library and (in step 13) which input and output libraries constitute one replicate.
  Steps 7 to 12 must be executed for each library, i.e., twice per replicate, replacing your_lib first with your_replicate‐in and then with your_replicate‐out.

### Trimming and UMI extraction (per library)

**Figure 2:**
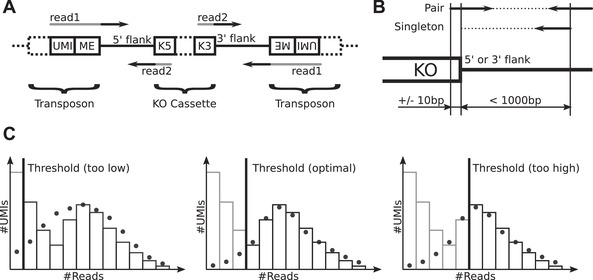
(**A**) Layout of sequenced fragments on both sides of knockout (KO) cassette insertions. The gray parts of the reads are non‐genomic and are trimmed before mapping. The unique molecular identifier (UMI) consists of 12 random bases, ME=5′‐AGATGTGTATAAGAGACAG‐3′. (**B**) Required mapping locations and directions for read pairs with either both mates or one mate mapped to be assigned to a specific KO. (**C**) Data indicating that the chosen TRUmiCount threshold is too low (left), optimal (middle), or too high (right).

7 To trim the technical sequences (Fig. 2A) from the raw sequencing reads for library *your_lib*, do
snakemake data/*your_design*/*your_lib*.trim.1.fq.gz
  Executing the trimming step for either read1 (in the command above) or read2 automatically trims the other read as well. If you started from an unmapped BAM file, the file is also automatically converted into two FASTQ files, with one for each read, during this step.
8 Optional (but recommended): To verify the trimming, generate FastQC (Andrews, 2010) reports for trimmed first and second reads with
snakemake data/*your_design*/*your_lib*.fastqc.1.html
snakemake data/*your_design*/*your_lib*.fastqc.2.html

### Mapping and assignment to insertional KOs (per library)

9 To map and assign trimmed reads to KOs (Fig. 2B) for the sequencing library *your_lib*, do
snakemake data/*your_design*/*your_lib*.assign.bam

### Determining KO abundances (per library)

10 To produce a table of genome abundance estimates for sequencing library *your_lib*, do
snakemake data/*your_design*/*your_lib*.count.tab
  For a description of the columns of data/your_design/your_lib.count. tab, see Table 2. During this step, the TRUmiCount algorithm (Pflug & von Haeseler, 2018) also produces the accompanying PDF report data/your_design/your_lib.count.pdf.

**Table 2:** Columns in the Knockout Abundance Table Generated by TRUmiCount in Basic Protocol 4, step 10

Column name	Type	Description
*gene*	String	Combination of knockout name and flank, “*knockout*:*flank*”
*n.umis*	Integer	Observed UMI count (after filtering)
*n.tot*	Numeric	Est. number of total UMI count, *n.umis/(1‐loss)*
*efficiency*	Numeric	Estimated PCR efficiency
*depth*	Numeric	Avg. number of reads per UMI (including lost UMIs)
*loss*	Numeric	Est. fraction of lost (unobserved) UMIs
*n.obs*	Integer	Same as for *n.umis*
*n.reads*	Integer	Observed total read count (after filtering)
*n.umis.prefilter*	Integer	Observed UMI count before read‐count filter
*n.reads.prefilter*	Integer	Observed total read count before read‐count filter

### Adjusting the TRUmiCount phantom rejection threshold (per library)

11 To see if adjustment of TRUmiCount's read‐count threshold is necessary, check read‐count distribution plot in the TRUmiCount report data/*your_design*/*your_lib*.count.pdf.

12 Optional: If there is a clear overabundance of observed vs. predicted UMIs for read counts slightly larger than the threshold, increase threshold. If the predicted and observed numbers of UMIs agree well for read counts below the threshold, decrease threshold (Fig. 2C). To set a library‐specific threshold *T* for library *your_lib*, add the following lines to the “trumicount” block in cfg/config.yaml (be sure to match the indentation of the existing lines in that block):
‐ file: 'data/your_design/your_lib.*'
opts: '‐‐threshold T'
  IMPORTANT NOTE: After changing the setting, remove data/your _design/your_lib.count.tab and re‐run step 10.

### Finding differentially virulent KOs (per replicate)

13 To compute the log fold changes of KO virulence for *your_replicate* and p‐values for how significantly these log fold changes deviate from zero (i.e., no change compared to the neutral reference set), do
snakemake data/your_design/your_replicate.dv.tab
  The differential virulence analysis is based on the KO abundances and loss correction factors from the tables your_replicate‐in.count.tab and your_replicate‐out.count.tab, both created in the folder data/your_design during step 10. Step 13 also produces an accompanying HTML report in data/your_design/your_replicate.dv.html.

### Downstream analysis

14 To produce plots and to combine data from multiple replicates, load output table data/*your_design*/*your_replicate*.dv.tab from step 13 into a table‐oriented tool such as Microsoft Excel, GraphPad Prism, R, or SPSS. Then, filter KOs based on fold change, p‐value, q‐value, number of genomes, etc. Combine data from multiple replicates either by filtering based on criteria from multiple replicates (e.g., significance) or by computing a combined p‐value (e.g., with Fisher's method).
  See Table 3 for a description of the columns of data/your_design/your_ replicate.dv.tab, which contains the results of the differential virulence analysis step.

**Table 3:** Columns in the Differential Virulence Table Generated in Basic Protocol 4, step 13

Column name	Type	Description
*knockout*	String	Name of the knockout as in the knockout list GFF file
*is.neutral*	Flag	1 if the knockout is part of the reference set, 0 otherwise
*n.out*	Integer	Sum of 5′ and 3′ UMI counts after filtering (output pool)
*loss.out*	Numeric	Average of 5′ and 3′ est. fraction of lost UMIs (output pool)
*abundance.out*	Numeric	Est. number of genomes, 0.5*n.out/(1‐loss.out) (output pool)
*n.in*	Integer	Sum of 5′ and 3′ UMI counts after filtering (input pool)
*loss.in*	Numeric	Average of 5′ and 3′ est. fraction of lost UMIs (input pool)
*abundance.in*	Numeric	Est. number of genomes, 0.5*n.in/(1‐loss.in) (input pool)
*log2fc*	Numeric	Virulence log fold change compared to the reference set (log_2_ ∆v)
*low.pval*	Numeric	p‐value for *log2fc* being significantly low
*high.pval*	Numeric	p‐value for *log2fc* being significantly high
*low.qval*	Numeric	FDR‐corrected p‐value for log2fc being significantly low
*high.qval*	Numeric	FDR‐corrected p‐value for log2fc being significantly high

## REAGENTS AND SOLUTIONS

### B&W buffer, 1×

5 mM Tris, pH 7.5
1 M NaCl
0.5 mM EDTA
Store ≤6 months at room temperature

### B&W buffer, 2×

10 mM Tris, pH 7.5
2 M NaCl
1 mM EDTA
Store ≤6 months at room temperature

### Potato dextrose–agar plates containing 200 µg/ml hygromycin

2.4% (w/v) potato dextrose broth (Difco)
2.0% (w/v) agar (Difco)
Add sterile deionized water and autoclave at 121°C
Cool to 50°C, add 50 mg/ml hygromycin (Roche) to 200 µg/ml, and mix by stirring
Pour 20 ml per 9‐cm petri dish
Store ≤2 weeks at 4°C in the dark

### Reassociation buffer

10 mM Tris, pH 8.0
50 mM NaCl
1 mM EDTA
Store ≤6 months at room temperature

### TAPS buffer, 5×

250 mM TAPS‐NaOH
125 mM MgCl_2_
50% (v/v) DMF
Adjust to pH 8.5 at 25°C
Store ≤4 weeks at room temperature in the dark in a cabinet for flammable liquids

CAUTION: DMF is toxic; work in a laminar flow hood with appropriate protective measures.

### Ustilago lysis buffer

10 mM Tris, pH 8.0
100 mM NaCl
1 mM EDTA
2% (v/v) Triton X‐100
1% (w/v) SDS
Store ≤6 months at room temperature

### YepsLight liquid medium

1.0% (w/v) yeast extract (Difco)
0.4% (w/v) Bacto Peptone (Difco)
0.4% (w/v) sucrose
Add sterile deionized water and autoclave at 121°C
Store ≤4 weeks at room temperature

## COMMENTARY

### Background Information

The analysis of insertional mutant libraries is well established for bacteria (Gawronski et al., 2009; Goodman et al., 2009; Langridge et al., 2009; van Opijnen et al., 2009) but has not been applied extensively to eukaryotic microorganisms. Genome‐wide insertional mutant libraries were generated successfully for baker's yeast (*Saccharomyces cerevisiae*) by homologous recombination (Winzeler et al., 1999) and, more recently, by transposition (Michel et al., 2017) and for the rice pathogenic fungus *Magnaporthe oryzae* by the kinase ATM (Jeon et al., 2007). However, tools that allow for efficient high‐throughput analysis of a negative depletion screen in the context of a host, for instance in the case of *M. oryzae* and rice (Jeon et al., 2007), were not available until recently. Therefore, we developed iPool‐Seq, which, due to its high selectivity and sensitivity, allows for analysis of pooled infections of insertional mutants directly from the infected host tissue (Uhse et al., 2018). iPool‐Seq provides a powerful tool for scientists who want to analyze mutant pool composition after colonization of the host, without any biases that could arise due to separation of the output fraction.

#### Outlook

We previously demonstrated (Uhse et al., 2018) that iPool‐Seq offers an elegant possibility to analyze a *U. maydis* insertional mutant pool after colonization of its host maize. We further suggest that iPool‐Seq could be applied to genome‐wide insertional mutant pools generated by high‐throughput techniques, e.g., transposon‐mediated mutagenesis. Due to its high selectivity and sensitivity, iPool‐Seq enables analysis of large insertional mutant pools directly from the colonized host tissue and obviates the need for separation of the host tissue and colonizing microbes. We propose that iPool‐Seq not only is suitable for analysis of mutant pools of microorganisms in the context of a plant host but also may be applied to animal‐microbe or microbe‐microbe interaction systems.

#### Trimming and UMI extraction

During this step, all non‐genomic sequences (with UMI and KO cassette overlap) are removed from the raw sequencing read pairs, as produced by (paired‐end) sequencing (Fig. 2A), so as to not interfere with the mapping process. The UMIs are instead stored as part of the pairs’ read names to ensure that the UMIs are passed alongside the reads through the following processing steps. Reads that do not overlap with the KO cassette or that do not contain a UMI are removed.

#### Mapping and assignment to insertional KOs

During this step, the trimmed reads are mapped to the reference genome using NextGenMap (Sedlazeck, Rescheneder, & von Haeseler, 2013) and assigned to the individual insertional KOs (Fig. 2B). In short, proper read pairs (pairs with both mates mapped and correctly oriented) are assigned to a specific flank (5′ or 3′) of a KO cassette insertion (i.e., a KO strain) if one read starts close (±10 bp) to the respective end of the cassette and extends away from the cassette. Singleton reads (reads whose mate could not be mapped) must map ≤1000 bp away from the respective end of the cassette and extend toward the cassette. Reads that cannot be assigned unambiguously are not assigned at all.

#### Determining KO abundances (counting genomes)

This step consists of error correction, phantom removal, and loss estimation for the UMIs detected for a particular combination of flank and KO strain. The UMIs are first corrected for sequencing errors with UMI‐Tools (Smith, Heger, & Sudbery, 2017), which merges similar UMIs found within the same flank of a KO cassette insertion. The merged UMIs are then processed further with TRUmiCount (Pflug & von Haeseler, 2018), which filters based on per‐UMI read counts to remove additional *phantom UMIs* (mostly amplification artifacts) and then, for each flank of each KO cassette insertion, estimates and corrects for the percentage of lost (i.e., unobserved) true UMIs. This ensures that the estimated KO abundances are unaffected by PCR amplification bias. The output comprises, per combination of KO and flank, the filtered UMI count (number of observed genomes), the estimated loss, and the loss‐corrected genome count (Table 2).

### Critical Parameters

#### Generating insertion libraries suitable for iPool‐Seq

There are different techniques available for generation of insertion libraries. We based the generation of the *U. maydis* insertional mutant library on homologous recombination. Insertional mutagenesis via homologous recombination has the advantage of not being prone to multiple insertions per individual, which is more likely to happen with untargeted, random insertional mutagenesis approaches, like Agrobacterium‐mediated transformation (Michielse, Hooykaas, van den Hondel, & Ram, 2005). However, homologous recombination is more laborious than high‐throughput methods. Moreover, the choice of the fungal model system is critical, and advantages and drawbacks of systems should be evaluated prior to insertional mutant library generation. Here, we use *U. maydis*, a fungal model that is genetically accessible to homologous recombination and well suited for the application of iPool‐Seq in the context of a host infection due to the availability of a solo‐pathogenic strain (Bölker, Genin, Lehmler, & Kahmann, 1995).

For the final analysis (Basic Protocol 4), an internal reference set of mutants with unaffected virulence is essential to find mutants that are comparatively depleted or enriched mutants. The reference set can be defined either by known unaffected mutants that have been published before or by individual mutants identified via infection tests.

#### Mutant pool infection

The iPool‐Seq protocol provides a high specificity for the inserted sequences and thus can be applied directly to infected host material (Basic Protocol 1). However, the number of pathogens that infect an individual host can constitute a bottleneck. We suggest overcoming this bottleneck by increasing the number of infected host plants or by reducing the complexity of the insertion mutant library.

#### Sequencing

We recommend sequencing the finished library (Basic Protocol 3) on an Illumina MiSeq platform and aiming for 2 to 3 million reads per library. However, it is also possible to use a different platform and to sequence more deeply to improve individual mutant UMI counts.

For complex libraries, it is possible to increase the amount of gDNA for the infected output sample and to proportionally increase the sequencing depth, which will likely yield a higher coverage of individual mutants. For a given mutant library and amount of extracted gDNA, the average number of reads per UMI (found in the TRUmiCount report) is an indicator of whether increasing the sequencing depth would be beneficial. For libraries with <1 read per UMI on average, deeper sequencing can be expected to improve the accuracy of abundance measurements; after that, the benefit drops gradually, and more than ∼10 reads per UMI will not provide any additional benefit.

### Troubleshooting

#### Trimming and UMI extraction

The optional FastQC (Andrews, 2010) reports created for the first and second reads after trimming offer a first quality check of library preparation and sequencing (Basic Protocol 4). Aspects to check for are as follows: (a) under “Basic Statistics,” that most (>90%) reads survived the trimming step; (b) under “Per base sequence quality” and “Per base N content,” that the sequenced bases are high quality and do not contain many Ns; and (c) under “Adapter Content,” that trimming indeed removed all sequencing adapters from the reads. If many reads are lost during the trimming step, they either were contaminants or did not match the sequence pattern that the library preparation should produce. In this case, we recommend blasting a few random reads to check for contamination and to manually compare their sequence composition to the expected pattern (Fig. 2A) and to the sequences in data/*your_design*/cassette.fa . Should most reads survive but show either a strong drop‐off of base qualities toward the end or many Ns, it may be necessary to include an additional quality‐based trimming step in the Trimmomatic (Bolger, Lohse, & Usadel, 2014) command in cfg/config.yaml (see the Trimmomatic manual for details). If there are still adapter sequences detected in the trimmed reads, add any custom adapter sequences that differ from the adapters mentioned in Basic Protocol 3 to cfg/Uhse_et_al.2018.adapters.fa (again, see the Trimmomatic manual for details).

#### Mapping and assignment to insertional KOs

We recommend checking the results of the mapping and KO assignment process visually for a few libraries and KO cassette insertions in a genomic viewer like the Integrated Genomics Viewer (IGV; Robinson et al., 2011). You should find most of the reads mapped to the two flanks (3′ and 5′) of KO cassette insertions, carrying the name and flank of the insertional KO in the form “*name*:*flank*” in the XT tag, and not extending more than a few base pairs into the regions replaced by the KO cassette. You may find some spurious reads mapped to arbitrary locations in the genome; these will later be ignored by the pipeline and thus are not a cause for concern (unless overly abundant).

If reads are mapped correctly but not assigned to the correct KO cassette insertion, check that the genomic coordinates in your GFF2 files listing the KO cassette insertions are correct. If a substantial fraction of the sequenced fragments are longer than 1000 bp or if many reads extends more than a few base pairs into the regions replaced by the KO cassette due to mapping imprecisions, adjust the “mapping fuzzyness” or “max fragment length” parameter of the “knockout_assignment” step in cfg/config.yaml .

#### Adjusting the TRUmiCount phantom rejection threshold

For optimal separation of phantom UMIs (i.e., amplification artifacts) from true UMIs by the TRUmiCount algorithm (Pflug & von Haeseler, 2018), it can be necessary to adjust the automatically chosen read‐count threshold T (Fig. 2C). This is true in particular at higher sequencing depths, where *phantom UMIs* can make up a large proportion of UMIs (but not of reads, due to the phantom's lower read counts). Setting the threshold too low (Fig. 2C, left) will cause phantom UMIs to be mistaken for true UMIs, which has the potential to distort the results. It will also distort TRUmiCount's parameter estimates (the PCR efficiency in particular), resulting in a bad fit of model and data. Choosing a threshold that is too high (Fig. 2C, right), in contrast, will cause more *true UMIs* (i.e., UMIs that reflect actual genomes in the sequenced pool) to be filtered out. However, given that TRUmiCount estimates and corrects for this loss, the net effect is only a reduced precision of the measured abundances (due to the lower absolute genome counts), and not an introduction of systematic biases. When choosing a threshold value, too high is thus preferable over too low.

### Understanding Results

The results contain several types of information. Firstly, the KO abundance tables (produced in Basic Protocol 4, step 10) list the number of genomes per mutant found in the input and output libraries, i.e., they contain information about the absolute abundances of the individual KOs. These tables in particular also provide information about which mutants are not detected at all in either input or output (i.e., that show zero detected genomes), which is possibly due to very slow growth or not being viable at all. Gradual changes in a mutant's virulence are detected by comparison of the mutant's input and output abundances to a reference set of neutral mutants and summarized in the differential virulence report (produced in Basic Protocol 4, step 13).

#### Finding differentially virulent KOs (statistical analysis of abundances)

Given that KO abundances in the input can be spread across multiple orders of magnitude, the dominant factor that determines the abundance of a KO in the output pool is its abundance in the input pool; the effects due to different genotypes that we want to detect is typically subordinate to that. The iPool‐Seq pipeline accounts for this by assuming that a KO's loss‐corrected abundance A_out_ in the output pool depends (linearly) on its loss‐corrected abundance A_in_ in the input pool, in addition to depending on the virulence factor ∆v relative to neutral KOs (∆v = 1 for neutral KOs). To account for differences in genome capture efficiency and total genome count between the two libraries, the pipeline also includes a scaling factor λ (which is replicate‐specific, but not KO‐specific). The loss‐corrected input and output abundances of a KO are computed from the (3′ and 5′ summed) filtered UMI counts N_in_ and N_out_, which are corrected for unobserved UMIs by dividing by 1 − ℓ_in_ and 1 − ℓ_out_, where ℓ_in_ and ℓ_out_ are the (3′ and 5′ averaged) loss estimates computed by TRUmiCount. The pipeline thus computes the log_2_ fold change of a KO's virulence as

log2Δv=log2A out λ·A in =log2N out ·1−ℓ in λ·N in ·1−ℓ out

To test for significant deviations of ∆v from neutrality (i.e., ∆ v= 1), the pipeline assumes a negative binomial model for N_out_ given N_in_, which accounts for the uncertainty in A_out_ and A_in_ due to sampling noise, as well as for an additional amount of dispersion d due to random fluctuations of the virulence of neutral KOs:

N out |N in ∼ NegBin μ=λ·N in ·1−ℓ out 1−ℓ in ,r=N in 1+d·N in

The two parameters λ and d are estimated by fitting the model to the set of neutral KOs. Parameter λ measures the relative total size (i.e., the loss‐corrected number of UMIs) of the output library relative to the input library, and d the squared coefficient of variation of output abundances due to biological noise (i.e., due to differences in the growth of neutral mutants).

#### Interpretation of the differential virulence report

The differential virulence report created in Basic Protocol 4, step 13, contains diagnostic statistics and plots that serve as quality checks and as verification that the assumptions of the statistical model are fulfilled to a reasonable degree.

The “Quality Control / Read and UMI count statistics” provide an overview of how many usable reads and UMIs remain after each step of the analysis pipeline. Discarding up to about one third of reads during the course of the analysis should be considered normal; if considerably fewer reads than that remain after the “TRUmiCount” step, the steps that remove the largest percentages of reads should be checked carefully for problems. For the number of UMIs, at high sequencing depths (on average ∼10 reads per UMI or more), it is normal for the removed percentage to be considerably higher because of TRUmiCount filtering of UMIs with a low read count.

The precision of the KO abundances determined for the input and output pools is reflected by the correlation (found under “Quality Control / Correlation of 3′ and 5′ Flank Abundances”) of the abundances computed for each flank of the KO cassette insertions. After loss correction by TRUmiCount, a correlation of ∼0.9 or more can be expected.

For neutral KOs, our statistical model also assumes a linear relationship between pre‐infection input abundance and post‐infection output abundance, which can be verified in the input vs. output plots and correlations (found under “Quality Control/Correlation of Input and Output Abundances”). There exists no generally applicable lower bound for the expected correlation of input and output because the expected correlation depends on the percentage of non‐neutral KOs among the KOs in the experiment and on how much faster or slower these KOs proliferate. More important than the correlation is the qualitative behavior seen in the input vs. output plots: the relationship should be linear across the full range of observed input abundances, without any plateau effect for highly abundant KOs. If such a plateau effect is observed, it is likely that the carrying capacity of the host plants has been reached, and either the number of mutants that each plant is infected with should be reduced or the statistical model should be modified to account for the carrying capacity of the host plant.

### Time Considerations

The iPool‐Seq protocol can be finished in ∼3 weeks for the *U. maydis–Zea mays* pathosystem (Fig. 1). For other pathosystems, we recommend first determining critical parameters and generating an insertional mutant library for infection. In comparison to *U. maydis*, variations in time considerations for other pathogens will mainly depend on the infection protocol.
